# Contrasting extremism and radicalisation in a syndemic society

**DOI:** 10.12688/openreseurope.14494.1

**Published:** 2022-05-06

**Authors:** Francesco Antonelli

**Affiliations:** 1 Political Sciences, Roma Tre University, Roma, 00146, Italy

**Keywords:** Radicalisation, Terrorism, P\CVE, Syndemia

## Abstract

The purpose of this document is to make recommendations on the necessity to transform current P/CVE policies, emphasizing the importance of a more effective involvement of Politics to contrast the spread of extremism and radicalisation in Europe. In fact, in a syndemic and post-Caliphate world, problems and challenges toward political violence are more and more institutional, systemic, and political.

According to the first HORIZON2020 PARTICIPATION PROJECT’s results, the crisis of participation and the crisis of integration are closely linked to current extremism and radicalisation processes; at the same time, fragmentation, interrelation between different kind of extremism and hybridisation, request a new approach that involve, in a more effective way, civil society and political society to contrast extremism, radicalisation and terrorism.

## 1. Introduction

In the contemporary world, we face a new scenario that influences the spread of radical and extremism attitudes: the **syndemic global society**. This is a society characterised from an increasing interaction between the biological and the social in disease burden as well as a complex set of inequalities, economic and social imbalances, risks, and threats at both local and global level ( Horton, 2020; Singer *et al.*, 2017). 

The transformation of the Jihad arena toward an increasing fragmentation and inner unrest after the defeat of the Caliphate in Syria and Iraq, the spread of far-right extremism, the rekindling of the migration crisis, the increase of international tensions and, last but not least, the social-economic crisis and riots due to the coronavirus disease 2019 pandemic, are the main challenges with which the “syndemic syndrome” occurs in Europe. These challenges directly or indirectly put into question the quality of our democracy because they focus on the crisis of integration and the crisis of participation ( Antonelli, 2022).

Indeed, in this scenario, it is necessary to **re-think the approach toward extremism and radicalisation**. Such a rethinking must involve not just socio-technical systems ( *e.g*. law enforcement agencies, social work, local bureaucracies and so forth) but also politics and ruling classes. In general, policy and politics towards extremism and radicalisation have been changed in recent years, after some spectacular and massive terrorist attacks occurred ( Coolsaet, 2018; Githens-Mazer, 2012; Schmid, 2013). In addition, politics has delegated the task to contrast extremist to specific technical or bureaucratic agencies, depoliticising the problem. On the contrary, this open letter is based on three suggestions:

- First, countering extremism and radicalisation requires an understanding of both their socio-political meaning in each context and their main developmental trends. **Moving from a “reactive” and “limited” to an “anticipatory” and “systemic” approach**.
- Secondly, the coming crisis of our democratic societies is twofold: a crisis of participation and a crisis of integration. If we consider “extremism” as a set of mass socio-political attitudes toward a de-legitimation of the system, and “radicalisation” as a process that involves minorities pushing them to political violence, **the first one is the place where the double crisis manifests itself and the second one is the place of an abnormal and dysfunctional response to such a crisis occurs**.
- Thirdly, countering extremism and radicalisation, especially in a fragile and boiling society such as the syndemic one, requires a political and systemic response and not a simple restyling.

## 2. Methods

- A systematic review of the recent literature on the drivers of radicalisation and violent extremism based on a mixed research design. This study analysed 350 international papers and reports published between 2015 and 2021 with the aim to identify main development trends in current extremism and violent radicalisation ^1^.
- Comparative research on P/CVE policies and strategies, at national and local level, in 8 European Countries: United Kingdom, France, Italy, Belgium, The Netherlands, Romania, Greece, Portugal. In addition, a special focus was on EU policies. Desk research and interviews involving experts were the methodologies for this study ^2^.
- Integrated research (systematic review on scientific literature and 16 semi-structured interviews with experts) on counter and alternative narratives communication strategies developed in Europe between 2015 and 2021 ^3^.
- A study on the characteristics and the effectiveness of the main risk assessment methodologies such as ERG22+ and VERA-2r ^4^.

## 3. Main emerging trends in extremism and radicalisation in Europe

✓ **Extremism is more and more a cumulative phenomenon:**by cumulative extremism it is generally meant a radicalisation process that starts, or is amplified, as a reaction to exposure to, or contact with, an ideologically different kind of extremism. There is a “game of symmetry” rather recurring: Jihadism increases far-right; Regarding far-left extremism and anarchism, the literature highlights the role of confrontation/clashes with the police even more than with right-wing extremists ( Marinone & Farinelli, 2021).
✓ **Digital cultures and gamification play an important role. They have a significant impact on the dynamics of imitation-emulation and on the consolidation of a group identity.** In addition, the increased online presence during the COVID-19 pandemic and lockdowns, has created a fertile ground for, amongst others, radicalisation and the spread of **conspiracy theories** ( Marinone & Farinelli, 2021).
✓ **Radicalisation process should be understood as an event that occurs at the intersection between a personal trajectory and a permissive, or enabling, environment.** In this context, it is important underline that radicalisation is a **relational experience** that involves the construction of own social status: relative deprivation and perceived inequality are at the centre of the stage. Similarly, the inner circle (friends and relatives), and/or the extended family unit (kinship) are very important to increase or to decrease the influence of any extremism narrative ( Marinone & Farinelli, 2021).
✓ **Many radicalised young people suffer from a shared lack of acceptance from the society which they live in**. Thus, youth seek refuge, acceptance and understanding within social networks. These virtual places may become the hotbeds of concrete solidarity through which youngsters may consolidate their personal identity. Lately, Europe has seen an increase in youth mobilisation with groups such as ‘Generation Identity’ which are starting to attract more young people across the continent. Literature highlights that the youth are drawn to these groups by a **sense of empowerment, vengeance, and opportunity to get out of poverty**. While social economic factors play a very important role in driving youth to violent extremism, **political participation** is increasingly important to them, especially when they feel they are not heard ( Marinone & Farinelli, 2021).
✓ **Assembling new kind of extremism (hybridisation):** this process takes place both between 'consolidated' extremisms and through the interaction with emerging phenomena such as, for instance, the proliferation of **conspiracy theories** or new religious movements (related to the recovery of an old tradition, such as Nordic mythology, or connected to more contemporary forms of spirituality, such as the New Age). **The coronavirus pandemic has certainly provided a very fertile ground for the proliferation of some of these new forms of extremism.**The literature highlights the emergence of a phase of re-assembly of the far right, which affects cultural imaginaries as well as the more complex construction of subjectivity. Less frequently mentioned in the literature, but in line with what has been stated so far, is the hybridisation between the extreme left and environmental extremism ( Antonelli, 2022; Marinone & Farinelli, 2021).

## 4. Preventing and countering violent extremism in Europe: general characteristics and criticalities

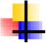
 **Islamist extremism** has remained a priority for several countries. This centrality can be found in risk assessment tools and strategies, used in prison and at local level around Europe ( do Céu Pinto Arena, 2021).

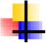
 **The focus on the social environment**. All European countries have displayed at least some understanding of the need to focus on the drivers of radicalization at a societal level. This includes educational initiatives and the training of civil society to act on a local level. In Belgium, the UK, and The Netherlands, for example, programmes focused on tackling youth unemployment and social polarization. Even in Italy where there is a large prioritization of law enforcement approach, there is a focus on civil society and education in preventing terrorism ( do Céu Pinto Arena, 2021).

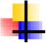
  A key part of preventing and countering violent extremism is the **information exchange and communication** between stakeholders and the public. Several policies have focused on this dimension and have created initiatives to engage the public in decision making. However, as particularly in the case of Belgium, even where plans are established to enable this communication, **participation** does not include members of the public, with the notable exception of Muslim communities ( do Céu Pinto Arena, 2021; Musolino, forthcoming).

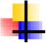
  A major challenge for eventual policy was the need to design a **coherent approach** to preventing and countering violent extremism and to avoid fatigue caused by introducing too many initiatives on different levels ( do Céu Pinto Arena, 2021).

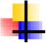
 **Risk assessment tools and methodologies are excessively linked to the prison, to the male and the adult populations**. They are inadequate when it is necessary to analyse the pre-criminal space, young people, women or alienated and discriminated ethnic, religious, sexual, and social minorities ( Manenti & Di Liddo, forthcoming).

## 5. Recommendations

According to the previous analysis, following recommendations can be suggested:

- **To contrast mass extremism**, in a preventive perspective, involving different actors, “civil society” and “politics society”, is the best way to contrast violent radicalisation and violent minorities, because of “it removes the water where fishes swim”.
- In a syndemic and post-caliphate world, re-thinking preventing and countering violent extremism systems is necessary, putting in the centre not just a single kind of extremism and radicalism, but a set of possible extremisms: it must move on from a one-dimensional approach, Jihad-centred, to **a multiple approach, centred on complexity, fragmentation, and hybridisation**.
- Put in the centre the awareness of **interrelation between different kind of extremisms**: the increase of one kind of extremism tends to increase other forms of extremism.
- Behind current extremism and radicalisation processes, there is **a demand for identity and subjectivation**. Consequently, at socio-political level, people request **more political participation and more social integration**, particularly young people living in marginal area or with low social status; extremism and radicalisation are often the result of a lack of both. All political actors must be aware on these critical issues.

## Underlying data

No data are associated with this article.
