# Supplementary material for: Contrasting extremism and radicalisation in a syndemic society
Source: Open Res Eur. 2022 May 6;2:54. [Version 1] doi: 10.12688/openreseurope.14494.1 (PMC10445815; doi:10.12688/openreseurope.14494.1)
Supplement: Peer review history

# Peer review: Reviewer response for version 1

**DOI:** 10.21956/openreseurope.15647.r29648

**Published:** 2022-07-29

**Authors:** Elisa Orofino

**Affiliations:** 1 The Policing Institute for the Eastern Region (PIER), Anglia Ruskin University, Chelmsford, UK

This open letter sheds light on the main emerging trends in extremism and radicalisation in Europe. This paper highlights the importance of “cumulative extremism” as the result of the interaction of different ideologies that fuel extreme views both online and offline. The author stresses the role of gamification in the online eco-systems as well as the importance of personal experience in conjunction with a fertile ground for the development of radical and extreme views.

 Besides showing an in-depth analysis of current knowledge on the phenomenon, this paper also points out the weaknesses of current risk assessment methods as being mostly related to the prison environment, to male and adult populations.

 This paper stresses the need to include civil society (organisations) in the co-production of knowledge on the complexities of extremism and radicalisation pathways. The paper also insists on the importance of including social actors in the design of new risk assessment tools.

 The paper is well designed and puts in evidence the main findings of the project PARTICIPATION coordinated by the same author. The paper successfully outlines the main findings and provides useful recommendations to policy and decision makers. This study is clearly and accurately presented, it cites the current relevant literature but I believe more studies could have been cited to support the author’s claims, e.g. Allchorn, W. (2020) “Cumulative Extremism and the Online Space: Reciprocal Radicalisation Effects Between the Extreme Right and Radical Islamists in the UK.” In: Littler, M. & Lee, B. (eds.) Digital Extremisms: Readings in Violence, Radicalisation and Extremism in the Online Space. London:Palgrave MacMillan. ^1^

 The study design is appropriate and the work has academic merit as it provides a snapshot of the current trends of extremism in Europe while outlining one of the main problems exacerbating it, i.e. the marginalisation of social actors.

 Sufficient details on methods and analysis are provided although something seems missing. In the methods section **, **the author states: “This study analysed 350 international papers and reports published between 2015 and 2021” but it would help to have more details on how these papers were selected, and what were the criteria for inclusion/exclusion used. Many scholars have written on the drivers of radicalisation over the years, so why and how were these specific 350 papers selected?

 This paper also needs copy-editing by a professional English language editor before indexing.

 Besides the minor points raised, this study is an excellent contribution to current knowledge on extremism and radicalisation within the EU context and can be used as a point of reflection for future policies and practices in the EU and beyond.

Where applicable, are recommendations and next steps explained clearly for others to follow? (Please consider whether others in the research community would be able to implement guidelines or recommendations and/or constructively engage in the debate)

Yes

Does the article adequately reference differing views and opinions?

Yes

Are all factual statements correct, and are statements and arguments made adequately supported by citations?

Yes

Is the rationale for the Open Letter provided in sufficient detail? (Please consider whether existing challenges in the field are outlined clearly and whether the purpose of the letter is explained)

Yes

Is the Open Letter written in accessible language? (Please consider whether all subject-specific terms, concepts and abbreviations are explained)

Yes

Reviewer Expertise:

I am currently the Academic Lead for Research on Extremism and Counter-Terrorism at the Policing Institute for the Eastern Region (PIER @ Anglia Ruskin University) and extremism and counter-terrorism have been the focus of my research since 2011. I have researched these themes in different national scenarios (e.g. Italy, Egypt, Australia, the UK) and I have published extensively on extremism, vocal extremist groups, radicalisation, Muslims in the West and social movements. More on my publications expertise and media engagement can be found at:https://aru.ac.uk/people/elisa-orofino.

I confirm that I have read this submission and believe that I have an appropriate level of expertise to confirm that it is of an acceptable scientific standard.

## References

[1] : “Cumulative Extremism and the Online Space: Reciprocal Radicalisation Effects Between the Extreme Right and Radical Islamists in the UK.” In: Littler, M., Lee, B. (eds.) Digital Extremisms: Readings in Violence, Radicalisation and Extremism in the Online Space. *Palgrave MacMillan* .2020;

# Peer review: Reviewer response for version 1

**DOI:** 10.21956/openreseurope.15647.r29647

**Published:** 2022-07-20

**Authors:** Bartolomeo Conti

**Affiliations:** 1 Centre d’Etudes des Mouvements Sociaux (CEMS), School for Advanced Studies in the Social Sciences (École des hautes études en sciences sociales; EHESS), Paris, France

This open letter is clear and well structured. It presents the problem in an interesting way, clearly indicates the sources used and the general characteristics and criticism of violent extremism in Europe. The recommendations are also clear and well developed. The rationale for the open letter is provided in sufficient detail and the arguments are adequately supported by data.

 We can only regret that some concepts or links are not explained further, probably due to the brevity of the document. The notion of “syndemic global society” is stimulating, but would benefit from further elaboration and clarification, as in its brief formulation it seems to be all-encompassing, at the risk of explaining nothing. As a result, the link between the syndrome and the need to rethink extremism is not so obvious.

 Similarly, the idea of considering *“extremism as a set of mass socio-political attitudes toward a de-legitimation of the system, and “radicalisation” as a process that involves minorities pushing them to political violence”*, would benefit from further explanation, as this interpretation, while legitimate and interesting, is not necessarily the one retained in other studies.

 The word "permissive" in this sentence is also problematic: " *Radicalisation process should be understood as an event that occurs at the intersection between a personal trajectory and a permissive, or enabling, environment*”.

 Beyond these considerations, which are rather an invitation to further explain certain concepts or links, the paper is clear, well written, and stimulating.

Where applicable, are recommendations and next steps explained clearly for others to follow? (Please consider whether others in the research community would be able to implement guidelines or recommendations and/or constructively engage in the debate)

Yes

Does the article adequately reference differing views and opinions?

Partly

Are all factual statements correct, and are statements and arguments made adequately supported by citations?

Yes

Is the rationale for the Open Letter provided in sufficient detail? (Please consider whether existing challenges in the field are outlined clearly and whether the purpose of the letter is explained)

Yes

Is the Open Letter written in accessible language? (Please consider whether all subject-specific terms, concepts and abbreviations are explained)

Yes

Reviewer Expertise:

Prisons, radicalisation, violent extremism, violence, exit violence, integration

I confirm that I have read this submission and believe that I have an appropriate level of expertise to confirm that it is of an acceptable scientific standard.

# Peer review: Reviewer response for version 1

**DOI:** 10.21956/openreseurope.15647.r29645

**Published:** 2022-07-13

**Authors:** Augusto Giuseppe Gamuzza

**Affiliations:** 1 Department of Educational Sciences, University of Catania, Catania, Italy

The open letter presents the main results of a H2020 research project highlighting the main emerging trends in radicalization studies through a multi-faceted approach (literature on the drivers of radicalisation and violent extremism; comparative research on P/CVE policies and strategies; systematic review on scientific literature and 16 semi-structured interviews with experts on counter and alternative narratives; risk assessment methodologies).

 The author presents the main retrieved outcomes, in comparative terms, with a good degree of clarity, unveiling relevant emerging trends (such as a multiple approaches, centred on complexity, fragmentation, and hybridisation) to design new approaches to radicalization in present times as well as stimuli for policies and P/CVE strategies. It is interesting to note the cross-validation of the main area of interest through multiple streams of information (i.e. academic and grey literature, interviews with experts). The recommendation section contains useful elements to consider as a logic consequence of premises and the reported analytic workflow.

Where applicable, are recommendations and next steps explained clearly for others to follow? (Please consider whether others in the research community would be able to implement guidelines or recommendations and/or constructively engage in the debate)

Yes

Does the article adequately reference differing views and opinions?

Yes

Are all factual statements correct, and are statements and arguments made adequately supported by citations?

Yes

Is the rationale for the Open Letter provided in sufficient detail? (Please consider whether existing challenges in the field are outlined clearly and whether the purpose of the letter is explained)

Yes

Is the Open Letter written in accessible language? (Please consider whether all subject-specific terms, concepts and abbreviations are explained)

Yes

Reviewer Expertise:

Sociology of terrorism and collective action, Methodology of social research,

I confirm that I have read this submission and believe that I have an appropriate level of expertise to confirm that it is of an acceptable scientific standard.
